# Long-term HIF-1α stabilization reduces respiration, promotes mitophagy, and results in retinal cell death

**DOI:** 10.1038/s41598-023-47942-8

**Published:** 2023-11-23

**Authors:** Nana Yaa Nsiah, Autumn B. Morgan, Nina Donkor, Denise M. Inman

**Affiliations:** 1 Department of Pharmaceutical Sciences, North Texas Eye Research Institute, University of North Texas Health Science Center, Fort Worth, TX USA; 2 Present Address: Genentech, South San Francisco, CA USA

**Keywords:** Visual system, Retina

## Abstract

Ocular hypertension during glaucoma can lead to hypoxia, activation of the HIF transcription factors, and a metabolic shift toward glycolysis. This study aims to test whether chronic HIF activation and the attendant metabolic reprogramming can initiate glaucoma-associated pathology independently of ocular hypertension. HIF-1α stabilization was induced in mice for 2 and 4 weeks by inhibiting prolyl hydroxylases using the small molecule Roxadustat. HIF-1α stabilization and the expression of its downstream bioenergetic targets were investigated in the retina by immunofluorescence, capillary electrophoresis, and biochemical enzyme activity assays. Roxadustat dosing resulted in significant stabilization of HIF-1α in the retina by 4 weeks, and upregulation in glycolysis-associated proteins (GLUT3, PDK-1) and enzyme activity in both neurons and glia. Accordingly, succinate dehydrogenase, mitochondrial marker MTCO1, and citrate synthase activity were significantly decreased at 4 weeks, while mitophagy was significantly increased. TUNEL assay showed significant apoptosis of cells in the retina, and PERG amplitude was significantly decreased with 4 weeks of HIF-1α stabilization. A significant increase in AMPK activation and glial hypertrophy, concomitant with decreases in retinal ganglion cell function and inner retina cell death suggests that chronic HIF-1α stabilization alone is detrimental to retina metabolic homeostasis and cellular survival.

## Introduction

Glaucoma is a complex neurodegenerative disease characterized by retinal ganglion cell loss and optic nerve neuropathy^1^. It is well-established that glaucoma is associated with hypoxia and HIF-1 activation. Immunohistochemical analysis of retinas from humans and preclinical animal models of glaucoma have revealed increased levels of HIF-1α relative to healthy controls^2–6^. HIF-1α protein coincided with areas of visual defects in retinas from human glaucoma patients^2^, associating HIF-1 activity with neurodegeneration in glaucoma. HIF-1 is a heterodimeric transcription factor usually activated under hypoxic conditions where it mediates metabolic adaptation to low oxygen availability by promoting glycolysis and limiting oxidative phosphorylation^7,8^. HIF-1α is rapidly degraded during normoxic conditions by oxygen-dependent prolyl-4-hydroxylases (PHDs). During hypoxia, HIF-1α protein accumulates because PHDs become inactive via iron insufficiency or 2-oxoglutarate depletion^9–11^. HIF-1α activity promotes the transcription of glucose transporters, glycolytic enzymes, and pyruvate dehydrogenase kinase genes, ensuring cells reliant on oxidative phosphorylation increase glycolysis to meet their energy needs^12^.

Previous studies by our group demonstrated high levels of HIF-1α in acute and chronic mouse models of glaucoma. Using magnetic beads injected into the anterior chamber to induce ocular hypertension (OHT), we showed HIF-1α activation within 6 h of OHT through to 4 weeks in many cells of the inner retina^13^. In the DBA/2J chronic glaucoma mouse model, we observed hypoxia in the retinal ganglion cell layer of 6- (onset of intraocular pressure elevation) and 10- (confirmed IOP elevation) month-old mice^14^. Altered glucose (primary substrate of glycolysis) and pyruvate (end-product of glycolysis) levels have also been reported in the retinas of the 9-month-old DBA/2J mice with high intraocular pressure (IOP) but no measurable signs of retinal ganglion cell (RGC) loss^15^, suggesting long-term HIF-1α associated metabolic reprogramming.

HIF-1 activation has been reported under normal oxygen conditions, a condition referred to as pseudohypoxia^16^. Pseudohypoxia is linked to pathological processes in aging, diabetes, and cancer^16–18^. We have demonstrated pseudohypoxia in the DBA/2J mouse and after induced OHT through a mismatch between HIF-1α stabilization and the expected promotion of glucose transporters (GLUTs), monocarboxylate transporters (MCTs), and lactate dehydrogenase A (LDHA)^12^. Induced OHT and DBA/2J mouse retina exhibit high levels of HIF-1α, yet unaltered levels of LDHA and downregulation of the glucose and monocarboxylate transporters in the optic nerve^4,14,19^. Alteration of the stereotypical downstream effects of HIF-1α was confirmed through the demonstration of pseudohypoxia in the DBA/2J mouse that was accompanied by a breakdown in communication between the nucleus and mitochondria, thus promoting mitochondrial dysfunction^14^. Despite the evidence of pseudohypoxia in glaucomatous retinas and optic nerves^14,20^, there are unanswered questions about the size of the role of increased HIF-1α to glaucoma pathogenesis.

We hypothesize that chronic HIF-1α-induced metabolic reprogramming to glycolysis is unsustainable in the retina, evolving into a pseudohypoxic state (as we have shown), but also generalized metabolic dysfunction in the retina. We intend to demonstrate this can occur independently of OHT.

Here, we modeled HIF-1α stabilization under normoxic conditions by administering a PHD inhibitor, Roxadustat. Roxadustat is approved for use in China, Japan, and the European Union for the treatment of renal anemia because it stimulates erythropoiesis^21^. In the presence of physiological oxygen levels, inhibition of PHDs prevents the hydroxylation and subsequent degradation of HIF-1α, resulting in the accumulation of HIF-1α, activation of the HIF complex, and increased expression of HIF-target genes including those encoding GLUTs, MCTs, LDHA, and hexokinase^22^.

We tested whether chronic HIF-1α signaling alone results in metabolic dysregulation, potentially providing insight into the metabolic deficiencies observed in situations with chronic HIF-1α activation, including glaucoma. We found that HIF-1α stabilization resulted in impaired visual function, altered the expression of several glycolytic and mitochondrial proteins, increased mitophagy, and led to apoptosis in the ganglion cell and inner nuclear layers. The glial hypertrophy and activated AMPK after 4 weeks of Roxadustat treatment resemble the conditions observed in chronic HIF-1α activation in glaucoma.

## Results

### Hypoxia inducible factor-lα protein

**Figure 1:**
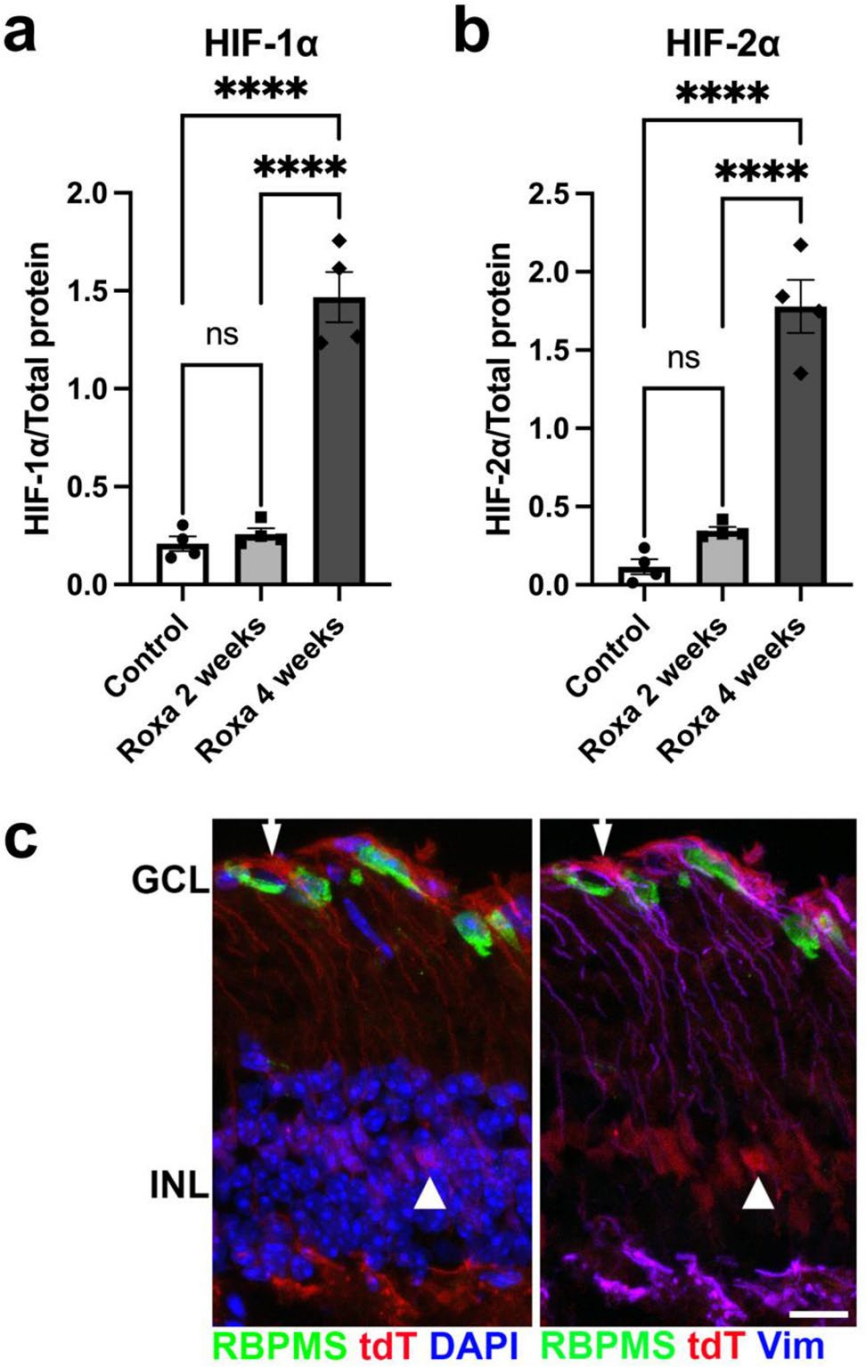
Verification that Roxadustat stabilizes HIF1-α in the retina. (**a**, **b**) Protein analysis of retinal lysates shows that I.P. injection of Roxadustat increased HIF-1α and HIF-2α protein in the retina. HIF-1α was significantly higher (F2,9 = 80, ****p < 0.0001) in 4-week Roxa retinas compared to 2-week Roxa and control retinas. HIF-2α was significantly higher (F2,9 = 77.23, ****p < 0.0001) in 4-week Roxa retinas compared to 2-week Roxa and control retinas. Tukey's multiple comparisons test showed a significant increase in HIF-1α and HIF-2α after 4 weeks of Roxa treatment (****p < 0.0001) compared to 2-week Roxa (****p < 0.0001) and control (****p < 0.0001) retinas. There was no difference in HIF-1α and HIF-2α in 2-week Roxa and control retinas (p = 0.8989, p = 0.7366, respectively). Values shown are mean ± SEM of **n = 4** per group. C) Retinal cross-section from CAG-CreERT2-ODD x ROSA26 flox’d STOP tdTomato mice 3 days after injection with Roxa and no OHT showing tdTomato (tdT, red) expression in cells with stabilized HIF-1α. RGCs (green) and Müller glia (blue, right panel) indicate that the primary tdTomato immunolabel is in Müller glial cell somata (arrowhead), and Müller glial basal processes (arrow). The right panel of (c) is the same view as the left, though showing Vimentin immunolabel in lieu of DAPI. GCL: ganglion cell layer; INL: inner nuclear layer. Scale bar: 25 μm.

Roxadustat stabilizes HIF-1α by inhibiting HIF prolyl hydroxylases, ultimately increasing HIF-1α protein levels. To confirm HIF-1α stabilization in the retina following systemic administration of Roxadustat, we analyzed retinal lysates for HIF-1α and HIF-2α protein by capillary electrophoresis. We observed a significant elevation in both HIF-1α and HIF-2α protein in mice treated for 4 weeks with Roxadustat (Roxa; see Methods for injection details) compared with control (~ sevenfold, p < 0.0001) and with mice treated for 2 weeks (~ fivefold, p < 0.0001), Fig. 1a and b. Two and four week time points were chosen to enable comparisons with studies of OHT and RGC structural and functional change^23,24^. Neither HIF-1α (Fig. 1a) nor HIF-2α (Fig. 1b) protein in 2-week Roxa-treated mice was significantly different from control mice (p = 0.8989 and p = 0.7366, respectively). Roxa treatment did not alter IOP; control mouse eye mean IOP was 13.4 ± 0.25, while 2-weeks of Roxa yielded IOP of 13.6 ± 0.28, and 4 weeks of Roxa treatment yielded IOP of 13.3 ± 0.25 mmHg (Supplementary Fig. 1 online). In addition to evaluating HIF protein levels, we used a hypoxia reporter mouse to also confirm that Roxa resulted in HIF-1α stabilization. CAG-CreERT2-ODD transgenic mice crossed to ROSA26 flox’d STOP-tdTomato mice were dosed for 5 days with tamoxifen to enable cre recombinase expression; three days following tamoxifen, mice were injected IP with Roxa. Cells with stabilized HIF-1α are labeled with a tdTomato reporter in these mice, and we found Roxa treatment (3 days) led to tdTomato expression in retinal glia, primarily Müller glia (Fig. 1c). These results show that PHD inhibition by systemic administration of Roxadustat successfully stabilized and elevated HIF-1α and HIF-2α in the retina.

### Roxa increases glycolysis and reduces glycolytic reserve

**Figure 2:**
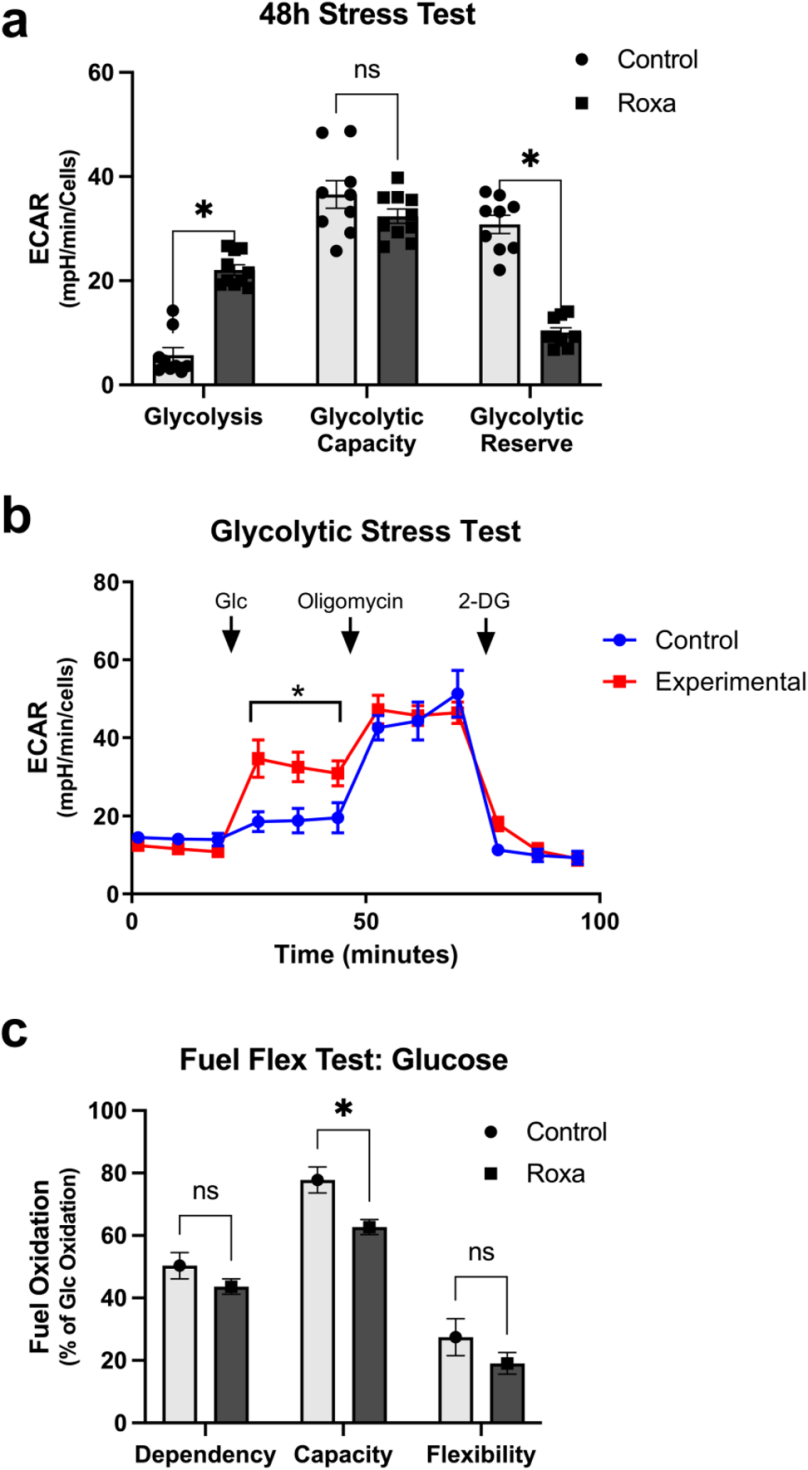
Roxadustat increases glycolysis in primary Müller glia. (**a**) Glycolysis, glycolytic capacity, and glycolytic reserve were calculated from the extracellular acidification rate (ECAR) in primary Müller glia exposed for 48 h to Roxadustat, as shown in (**b**). Müller glia exposed to Roxadustat engaged in significantly higher glycolysis as compared to control Müller glia (*p = 0.015); glycolytic capacity did not vary across the control and Roxa-treated cells. The Roxa-treated cells did exhibit significantly lower glycolytic reserve (*p = 0.02) than the control cells. (**b**) The moment-by-moment ECAR measures taken during the glycolytic stress test as glucose (Glc), then oligomycin, and finally, 2-deoxyglucose (2-DG) were sequentially added to the wells containing either control or Roxa-treated Müller glia. Glycolysis in the Roxa-treated cells was significantly higher than control cells (*p = 0.015). Graph shows example trace from a biological replicate. (**c**) Primary Müller glia subjected to a fuel flex test using glucose showed that the Roxa-treated Müller glia were no different from control in terms of their dependency on glucose or their flexibility towards glucose utilization, but they had significantly lower capacity than the control cells (*p = 0.028). Seahorse Bioanalyzer experiments were run with three separate primary Muller glial cell isolations, with **4–9** wells assessed per run for the data shown in **a** and **c**.

To confirm an impact of Roxadustat on glycolytic cellular metabolism, we cultured primary Müller glia, treated the cells with Roxadustat for 48 h, then used the Seahorse Bioanalyzer to examine glycolysis. Control and Roxa-treated Müller glia were subjected to a glycolytic stress test which yielded results indicating that Roxa-treated cells engaged in significantly higher levels of glycolysis than control cells (p = 0.015, Fig. 2a and b). Increases in glycolysis were anticipated due to the promotion of gene expression for glucose transporters and hexokinase due to HIF-1α activity^25^. The higher baseline glycolysis in Roxa-treated Müller glia led to significantly lower glycolytic reserve than control cells (p = 0.02, Fig. 2a). Figure 2b shows the extracellular acidification rate (ECAR) plot from the glycolytic stress test, which begins with the addition of glucose once baseline ECAR has been established, followed by the addition of oligomycin-A which inhibits ATP synthase in the cells; finally, the addition of the non-hydrolyzable 2-deoxyglucose halts all glycolysis-associated ECAR. We then used the Seahorse Bioanalyzer to investigate the dependency of the Roxa-treated Müller glia on glucose for fuel. Glucose dependency did not differ between the control and Roxa-treated Müller glia, nor did the flexibility of the cells’ use of glucose as a fuel. However, there was a statistically significant difference in the capacity of the Roxa-treated Müller glia to oxidize glucose (p = 0.028, Fig. 2c). This result differs from the capacity as calculated from the glycolytic stress test (Fig. 2a), possibly explained by the capacity measure being generated from ECAR values after oligomycin-A inhibition of the ATP synthase in Fig. 2a, while capacity in the fuel flex test (Fig. 2c) is measured from oxygen consumption rate after inhibiting the fatty acid and glutamine fuel pathways (carnitine palmitoyl-transferase 1A (CPT1A) using etomoxir and glutaminase using BPTES, respectively). Consult the Methods for the equation used. These analyses in primary Müller glia indicate that short-term inhibition of PHDs by Roxa increases glycolysis, but reduces glycolytic reserves and capacity.

### Effect of chronic HIF-1α stabilization on retinal energy generation

**Figure 3:**
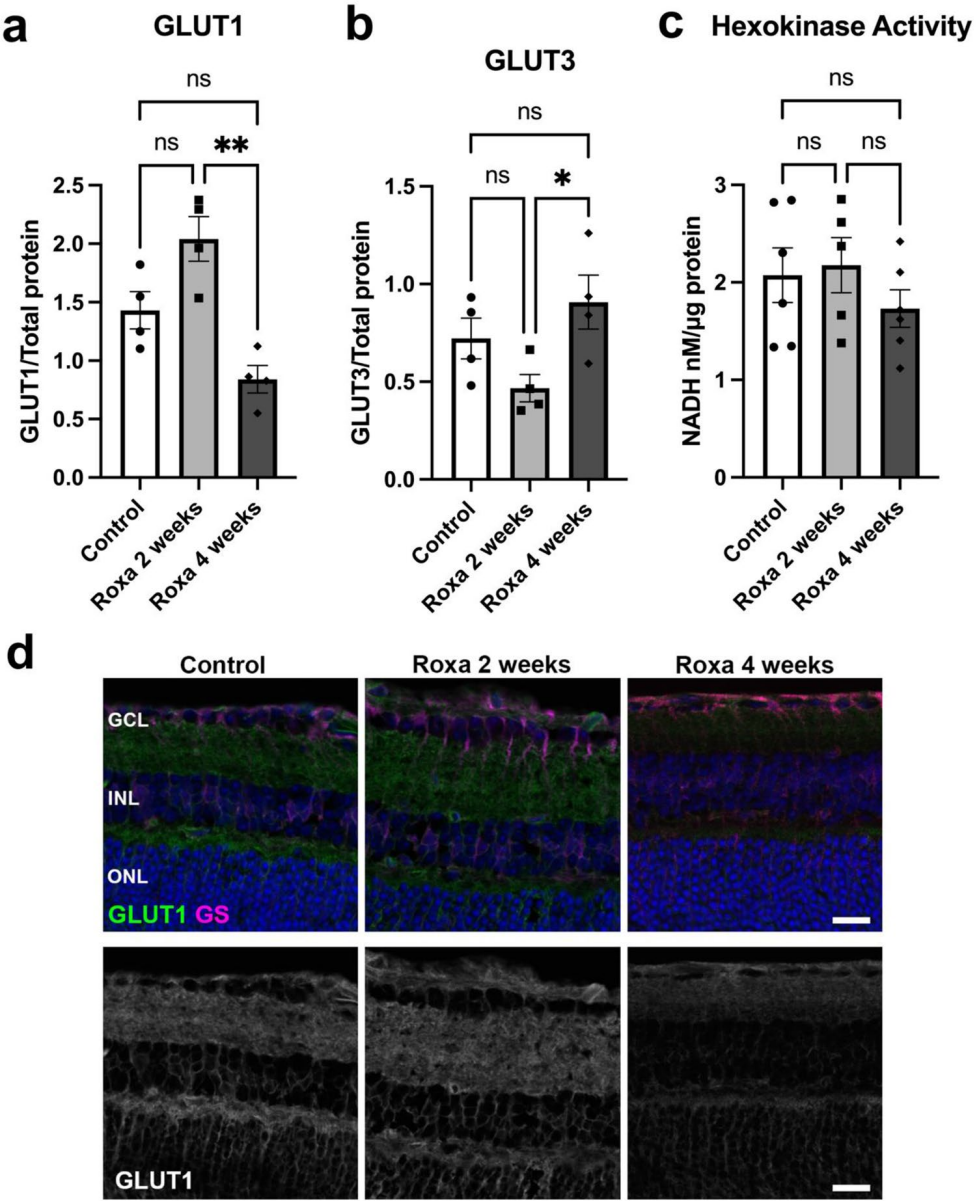
Glycolytic protein expression in the retina of control and Roxa-treated mice. (**a**) GLUT1 protein was significantly reduced (F(2,9) = 8.719, p = 0.0078) in 4-week Roxa retinas compared to 2-week and control retinas. Tukey's multiple comparisons test showed a significant reduction in GLUT1 in 4-week Roxa retina compared to 2-week Roxa retina (p = 0.0062). There was no difference in GLUT1 protein in control retina compared to 2-week Roxa (p = 0.1034) and 4-week (p = 0.2103) retinas. Values shown are mean ± SEM, **n = 4** samples per group. (**b**) GLUT3 protein was not different between control and Roxa-treatment groups (F(2,9) = 4.204, p = 0.0514). Tukey's multiple comparisons test showed GLUT3 in control retina was comparable to 2-week Roxa (p = 0.2688) and 4-week Roxa (p = 0.4714) retinas. 4-week Roxa GLUT3 protein was significantly higher than in 2-week Roxa retinas (p = 0.0430). Values shown are mean ± SEM of **n = 6** samples per group. (**c**) Hexokinase activity did not vary across groups (F(2, 14) = 0.8535, p = 0.4469), **n = 6** samples per group. Tukey's multiple comparisons test showed no difference in hexokinase activity between groups; control vs. 2-week Roxa (p = 0.9567), control vs. 4-week Roxa (p = 0.5974), and 2-week vs. 4-week Roxa (p = 0.4593). (**D**) Representative immunofluorescence images showing GLUT1 (green) and glutamine synthetase (magenta) protein levels in Control, Roxa 2-week, and Roxa 4-week retina cross-sections, Scale bar = 20 µm. GCL = ganglion cell layer; INL = inner nuclear layer; ONL = outer nuclear layer. Proteins were analyzed by capillary electrophoresis normalized with total protein levels in each capillary. Bar graph data presented as mean ± SEM, n = 4–6 per group.

Activated HIF-1α promotes glycolysis by increasing the expression of glucose transporter 1 (GLUT1), glucose transporter 3 (GLUT3), and hexokinase 1 and 2 genes^26^. To evaluate the effect of HIF-1α activation on glycolysis in the retina following Roxa treatment, we measured GLUT1 and GLUT3 protein levels and hexokinase activity. GLUT1 protein was higher but not statistically significant in the 2-week Roxa mouse retina compared with control (Fig. 3a). However, with chronic HIF-1α activation, GLUT1 protein was significantly reduced in retinas from 4-week Roxa mice compared with 2-week Roxa mice. GLUT1 protein in 4-week Roxa mouse retina was no different from control (Fig. 3a). GLUT3 protein was higher in the latter when comparing 2 and 4-week Roxa treatment, but 4-week GLUT3 protein levels were not different from the control (Fig. 3b). Hexokinase activity did not differ among control, 2-week, and 4-week Roxa treatments (Fig. 3c). Retinal cross-sections immunolabeled for GLUT1 and glutamine synthetase (GS) for Müller glia, which express GLUT1^27^, show the reduction in GLUT1 protein by 4 weeks of Roxa treatment (Fig. 3d).

### HIF-1α stabilization and lactate metabolism

**Figure 4:**
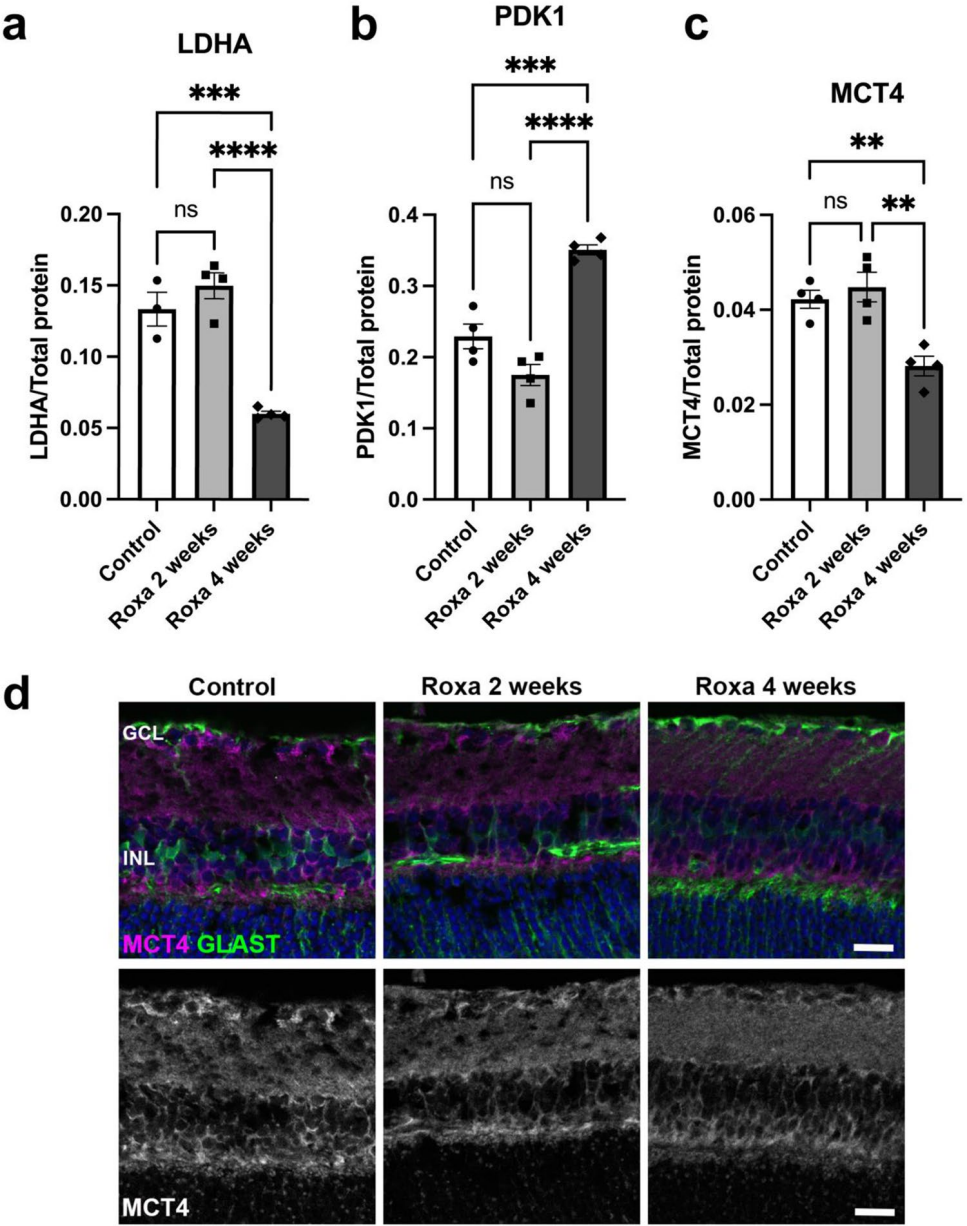
Effect of HIF-1α stabilization on LDHA, PDK1, and MCT4 protein expression. (**a**) LDHA protein was significantly reduced (F(2,8) = 38.55, p < 0.0001) in 4-week Roxa retina compared to control and 2-week retinas. Tukey's multiple comparisons test showed a significant reduction in LDHA in 2- week (p = 0.0006) and 4-week Roxa (p < 0.0001) retinas compared to control retinas. (**b**) PDK1 protein was significantly higher (F(2,9) = 42.73, p < 0.0001) in 4-week Roxa retina compared to control and 2-week retinas. Tukey's multiple comparisons test showed a significant PDK1 increase in 4 weeks compared to control (p = 0.0004) and 2-week Roxa (p < 0.0001) retinas. (**c**) MCT4 protein was significantly lower (F(2,9) = 13.64, p = 0.0019) in 4-week Roxa retina compared to control and 2-week retinas. Tukey's multiple comparisons test showed MCT4 decreased in 4 weeks compared to control (p = 0.0064) and 2-week Roxa (p = 0.0023) retinas. (**d**) Representative immunofluorescence images showing MCT-4 (magenta) and GLAST (green) expression in retina cross-sections for the Control, Roxa 2-week, and Roxa 4-week groups. Scale bar = 20 µm. GCL = ganglion cell layer; INL = inner nuclear layer. Proteins were analyzed by capillary electrophoresis and normalized to total protein levels. Values shown are mean ± SEM of **n = 3–4 per group**.

Besides promoting glycolysis, HIF activation inhibits the tricarboxylic acid cycle (TCA) and oxidative phosphorylation by preventing pyruvate entry into the TCA cycle. Specifically, HIF-1α induces the transcription of lactate dehydrogenase A (LDHA)^28^ and pyruvate dehydrogenase kinase 1 (PDK1)^29,30^. LDHA and PDK1 prevent the conversion of pyruvate derived from glycolysis to the TCA cycle metabolite acetyl-CoA. LDHA catalyzes the conversion of pyruvate generated from glycolysis to lactate, while PDK1 phosphorylates and inactivates pyruvate dehydrogenase, the enzyme complex that converts pyruvate to acetyl-CoA. Compared to control and 2-week Roxa mice, LDHA was significantly lower in 4-week Roxa-treated retinas despite high HIF-1α protein. There were no significant differences in LDHA protein levels in retinas treated with Roxa for 2 weeks and control mice (Fig. 4a). Consistent with HIF-1α protein accumulation and previous reports^29,30^, we observed significantly higher levels of PDK1 protein in 4-week Roxa mice compared to control and 2-week Roxa mice (Fig. 4b). Monocarboxylate transporters (MCTs) export lactate out of cells to avoid competitive inhibition of LDHA^31^. MCT-4 is expressed throughout the inner retina^32^, including Müller glia endfeet^33^. Quantifying MCT4, the predominant MCT upregulated in response to HIF-1α activation, we observed that MCT4 protein was significantly decreased in 4-week Roxa mice compared to control and 2-week Roxa despite HIF-1α accumulation (Fig. 4c and d). However, 2-week Roxa MCT4 protein levels were comparable to control mice (Fig. 4c). Retina cross-sections show diminished immunolabeling of MCT4 in the ganglion cell (GCL) and inner nuclear (INL) layers of retina (Fig. 4d).

### HIF-1α stabilization and oxidative phosphorylation

**Figure 5:**
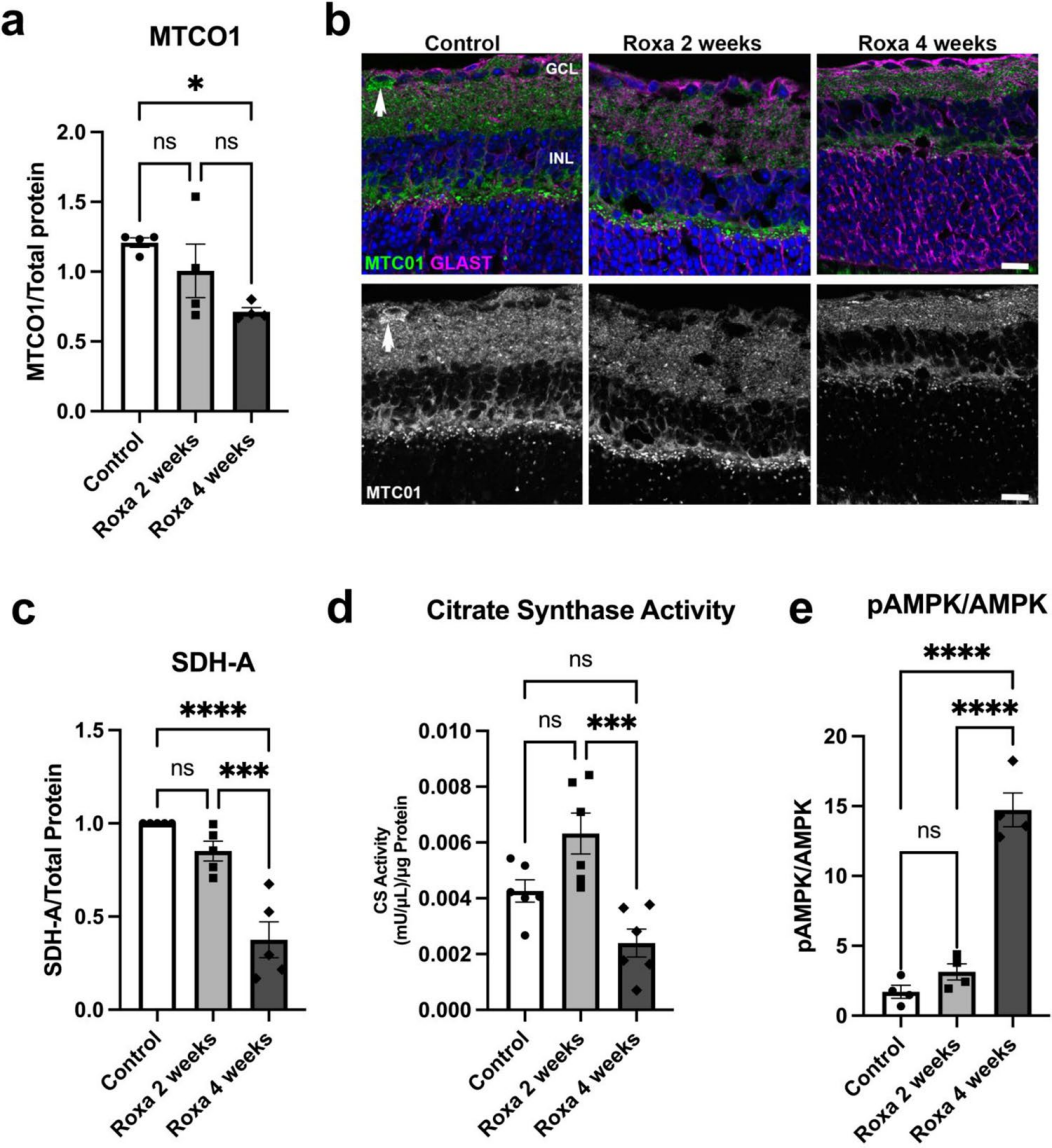
Chronic HIF-1α stabilization impairs mitochondrial function. (**a**) MTCO1 protein was significantly different between groups (F(2,9) = 4.814, p = 0.0379). Tukey's multiple comparisons test showed MTCO1 was significantly lower in 4-week Roxa retinas compared to control retinas (p = 0.0316). MTCO1 protein levels were not significantly different between control vs. 2-week Roxa (p = 0.4497) and 2-week vs. 4-week retinas; **n = 4** per group. (**b**) Mitochondrial cytochrome c subunit 1 (MTCO1, green) labels cytochrome c oxidase (COX) and GLAST (magenta) labels Müller glia. Arrow points to an RGC in the GCL with MTCO1 immunolabel. GCL = ganglion cell layer; INL = inner nuclear layer. Scale bar: 20 μm, (**c**) SDH-A protein was significantly lower (F(2,12) = 26.24, p < 0.0001) in Roxa-treated retinas compared to control. Tukey's multiple comparisons test showed SDH-A decreased in 4-week Roxa retinas (**n = 6**) compared to control (**n = 6**, p < 0.0001) and 2-week Roxa (**n = 6**, p = 0.0005) retinas. There were no differences in SDHA protein levels between control and 2-week Roxa retinas (p = 0.264). (**d**) Citrate synthase activity was significantly reduced in 4-week Roxa retinas (F(2, 15) = 12.26, p = 0.0007), **n = 6** samples per group. Tukey's multiple comparisons test showed citrate synthase activity decreased in 4-week Roxa compared to 2-week Roxa (p = 0.0005). There was no significant difference in citrate synthase activity in control vs. 2-week Roxa (p = 0.05) and control vs. 4-week Roxa (p = 0.0788). (**e**) The ratio of pAMPK to AMPK was significantly higher (F(2,9) = 76.26, p < 0.0001) in 4-week Roxa retina compared to control and 2-week retinas. Tukey's multiple comparisons test showed pAMPK to AMPK ratio increased in 4-week retinas compared to control (p < 0.0001) and 2-week Roxa (p < 0.0001) retinas; **n = 4** samples per group.

Given that HIF-1α impairs OXPHOS by downregulating electron transport chain proteins and promoting mitophagy, we measured cytochrome c oxidase and succinate dehydrogenase (SDH-A/Complex II) protein and citrate synthase enzyme activity. We found that Roxa treatment significantly decreased cytochrome c oxidase subunit 1 (MTCO1) in 4-week Roxa mice compared to control mice. We observed no significant differences in 2-week Roxa MTCO1 protein compared to control or 4-week Roxa mouse retina (Fig. 5a). However, a significant difference was observed between the control and 4-week mice (p = 0.0316). MTCO1 immunofluorescence (right panel of Fig. 5b) upholds the quantitative observations, with less MTCO1 protein in the 4-week Roxa treatment group. Retinal sections in Fig. 5b were immunolabled for MTCO1, but also GLAST for astrocytes and Müller glia, to provide context. MTCO1 immunolabel was concentrated in the IPL and the OPL, with immunolabel observed in an RGC in the Control retina (arrow). SDH-A protein was significantly reduced in the 4-week Roxa mouse retina as compared to the control (p < 0.0001)) and the 2-week Roxa treatment group (p = 0.0005; Fig. 5b). Citrate synthase activity was significantly lower in 4-week Roxa mice compared to the 2-week Roxa mouse retina (p = 0.0005; Fig. 5c) but no significant difference was observed compared to the control (p = 0.0788). The results indicate significant changes in mitochondrial respiration, mainly after 4 weeks of Roxa treatment. To obtain direct evidence of the negative impact of HIF-1 activation on energy generation, we examined AMPK activation because AMPK is activated in response to low cellular ATP levels. Consistent with the changes in glycolytic and OXPHOS proteins, we detected significantly higher pAMPK to AMPK ratio in the retina from 4-week Roxa mice as compared to control and 2-week Roxa mice (Fig. 5d).

### HIF-1α and Mitophagy

**Figure 6:**
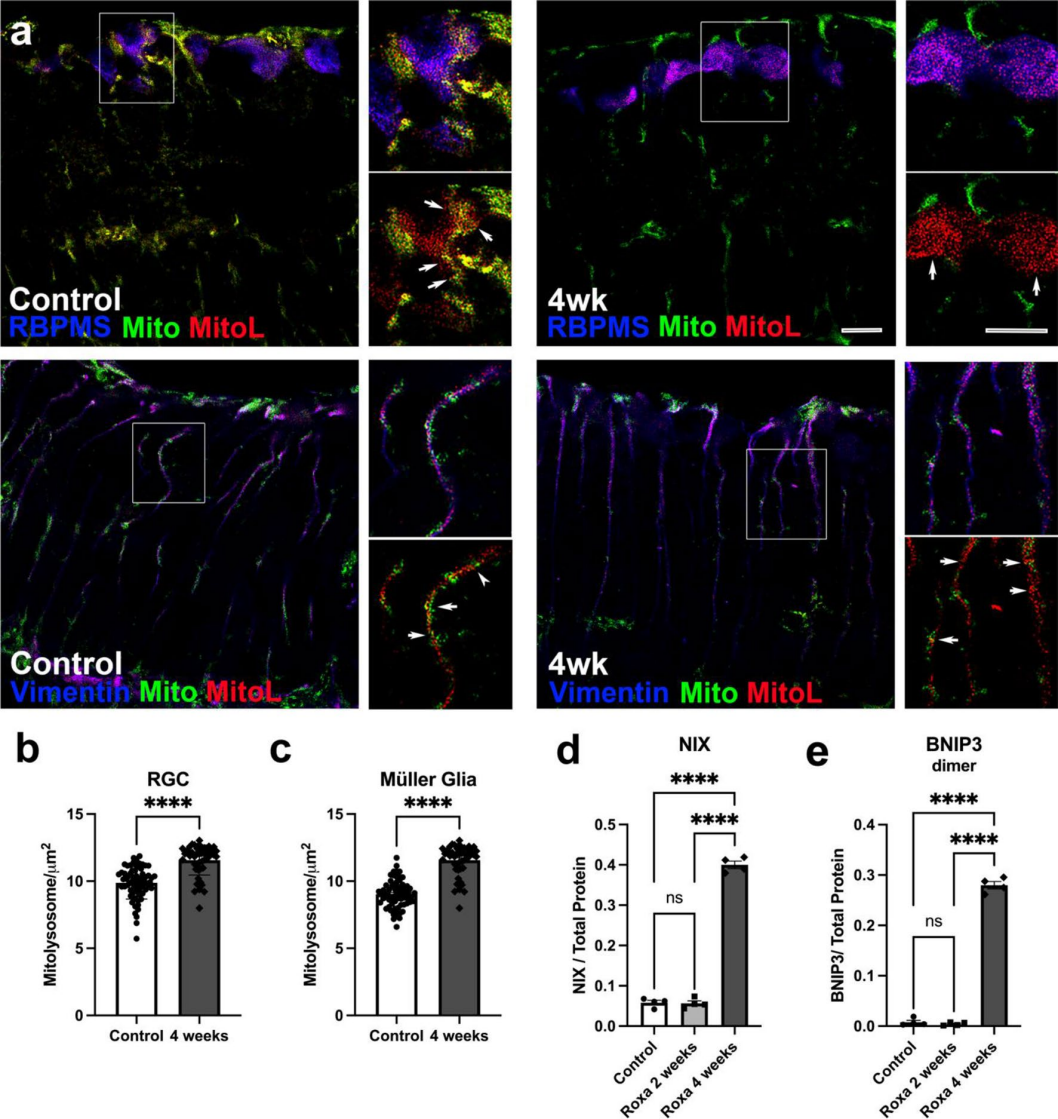
Roxa treatment upregulates mitophagy in RGCs. (**a**) Sections of control retina (left) and retina after 4 weeks of Roxa treatment (right) depict mitochondria (green and red, ‘Mito’) and mitolysosomes (red, ‘MitoL’) in RGCs immunolabeled for RBPMS (blue, top panels), and in Müller glia immunolabeled for Vimentin (blue, lower panels). Mitolysosomes in RGCs (**b**) and Müller glia (**c**) were significantly increased after 4 weeks of Roxa treatment; p < 0.0001 by Student’s t-test for RGCs (**b**) and Müller glia (**c**). Values are mean ± SEM of **n = 6** retinas per group. (**d**) NIX was significantly higher (F(2,9) = 759.6, p < 0.0001) in 4-week Roxa retinas compared to control and 2-week retinas. Tukey's multiple comparisons test showed NIX levels were significantly higher in 4-week retinas compared to control (p < 0.0001) and 2-week Roxa (p < 0.0001) retinas. There was no significant difference between control vs. 2-week Roxa retinas (p = 0.9848); **n = 4** per group. (**e**) BNIP3 dimer was significantly higher (F(2,9) = 1050, p < 0.0001) in 4-week Roxa retinas compared to control and 2-week retinas. Tukey's multiple comparisons test showed BNIP3 dimer levels were significantly higher in 4-week retinas compared to control (p < 0.0001) and 2-week Roxa (p < 0.0001) retinas. There was no significant difference between control vs. 2-week Roxa retinas (p = 0.8791). Values shown are mean ± SEM of **n = 4–6** per group. Proteins were analyzed by capillary electrophoresis and normalized to total protein levels.

To examine whether mitophagy may have contributed to the decrease in mitochondrial proteins, we subjected MitoQC mice to Roxa treatment (4 weeks) and assessed mitophagy in retinas sectioned and immunolabeled for RGCs using RBMPS and Müller glia using Vimentin (Fig. 6a). Mitophagy was evaluated by quantifying mitolysosomes in RGCs (Fig. 6b) and Müller glia (Fig. 6c). In these mice, the mitochondria carry green and red fluorescent proteins on their outer membranes. Upon engulfment of the organelles by a lysosome as part of mitophagy, the green fluorescence is quenched, leaving only the red fluorophore. The red puncta can be quantified to indicate the degree of mitophagy in the RGCs or Müller glia. We observed significant increases in mitolysosomes in both RGCs and Müller glia with four weeks of Roxa treatment (p < 0.0001; Fig. 6b and c). Under hypoxia, HIF induces mitophagy by upregulating the expression of several mitophagy regulators and receptors, notably Bcl2/adenovirus E1B 19 kDa protein-interacting protein 3 (BNIP3) and BNIP3L/NIX. BNIP3 exists as monomers and dimers; homodimerization is required for BNIP3 mitochondrial localization and activation^34–39^. As expected, BNIP3L/NIX and BNIP3 dimers were substantially upregulated in the retina after 4 weeks of Roxa treatment (p < 0.0001; Fig. 6d and e). BNIP3 monomer was not altered by Roxa treatment (data not shown). These results suggest that mitophagy may have contributed to the loss of mitochondrial and ETC proteins (MTCO1, SDH-A) and citrate synthase activity (Fig. 5) following chronic HIF stabilization in the retina.

### Reactive gliosis in the retina of Roxa-treated mice

**Figure 7:**
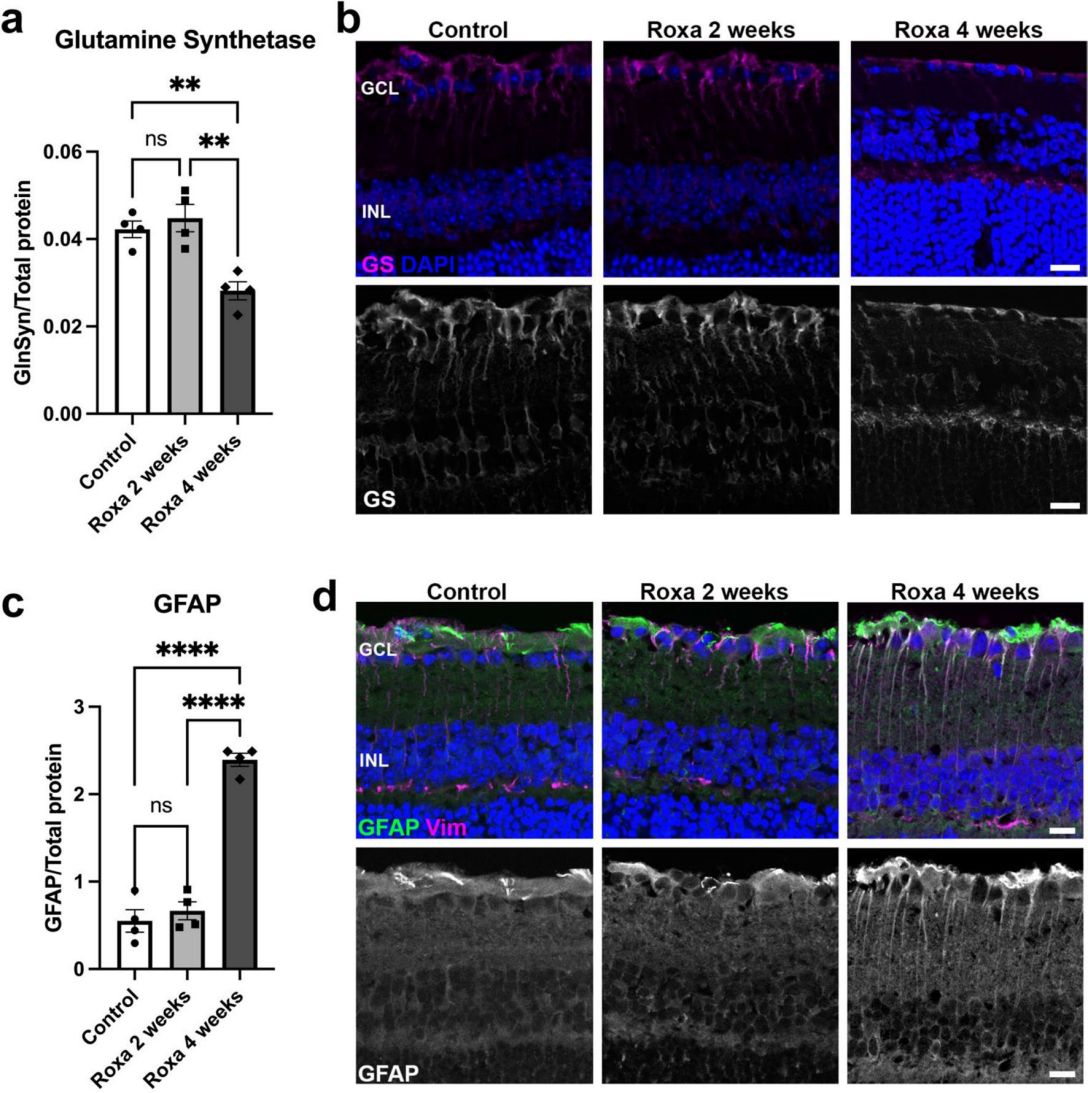
HIF-1α stabilization is associated with reactive gliosis. (**a**) GS protein was significantly lower (F(2,9) = 13.64, p = 0.0019) in 4-week Roxa retina compared to control and 2-week retinas. Tukey's multiple comparisons test showed GS decreased in 4-week Roxa compared to control (p = 0.0067) and 2-week Roxa (p = 0.0023) retinas. There were no significant differences in GS protein quantity in control vs. 2-week retinas (p = 0.7453). (**b**) Representative immunofluorescence image showing GS (magenta) on retinal cross-sections. (**c**) GFAP was significantly higher (F(2,9) = 1050, p < 0.0001) in 4-week Roxa retinas compared to control and 2-week retinas. Tukey's multiple comparisons test showed GFAP levels were significantly higher in 4-week retinas compared to control (p < 0.0001) and 2-week Roxa (p < 0.0001) retinas. There was no significant difference between control vs. 2-week Roxa retinas (p = 0.7241). (**d**) Immunofluorescence image showing GFAP (green) and vimentin (MG marker) on retinal cross-sections. GCL = ganglion cell layer; INL = inner nuclear layer. Scale bar: 20 μm. Values shown are mean ± SEM of **n = 4** per group.

Glial cell reactivity is frequently associated with retinal stress and neurodegeneration^31^. To examine whether glial cells were activated by chronic HIF-1 accumulation, we stained retinal sections for glutamine synthetase (GS) and glial fibrillary acidic protein (GFAP), a marker of glial activation (Fig. 7). Glutamine synthetase (GS) expressed by Müller glia is important for glutamate neurotransmitter recycling to prevent excitotoxicity. GS was significantly lower in 4-week Roxa retinas (Fig. 7a and b), while GFAP staining was upregulated compared to control and 2-week Roxa mice (Fig. 7c and d). In control and 2-week Roxa retinas, GFAP labeling was restricted to the nerve fiber layer (NFL), whereas, in 4-week Roxa retinas, GFAP staining extended from the NFL towards the inner nuclear layer (INL) (Fig. 7c and d). These data indicate that chronic HIF-1α elevation is associated with reactive gliosis and GS downregulation.

### Effects of chronic HIF-1α stabilization on RGC function

**Figure 8:**
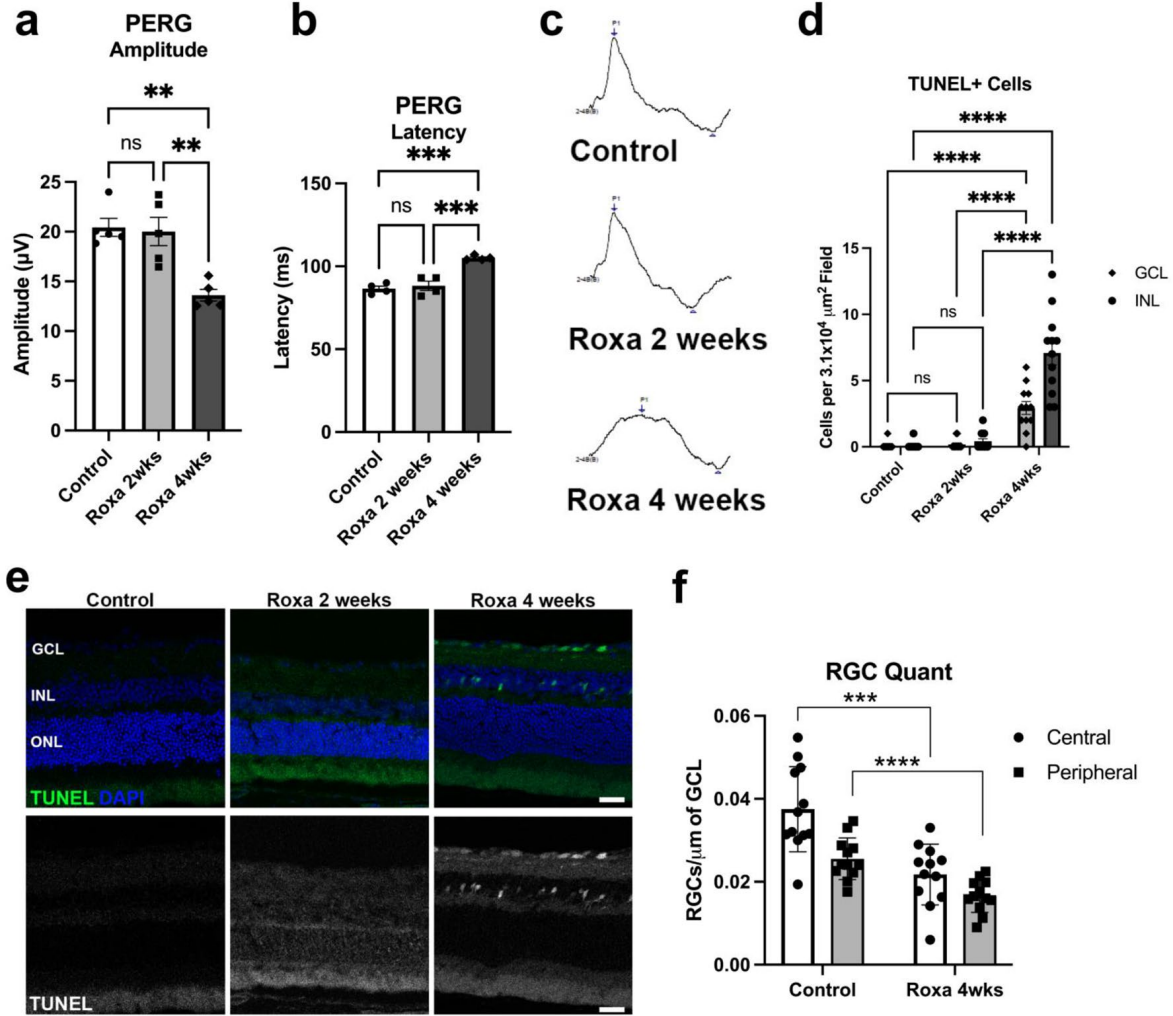
Chronic HIF-1α stabilization on RGC function. (**a**) PERG amplitude was significantly lower in 4-week Roxa mice compared to control and 2-week Roxa mice (F(2,12) = 13.64, p = 0.0008). Tukey's multiple comparisons test showed PERG amplitude decreased in 4-week Roxa compared to control (p = 0.0015) and 2-week Roxa (p = 0.0024) retinas. There were no significant differences in PERG amplitude between control vs. 2-week retinas (p = 0.9559). Values shown are mean ± SEM of **n = 5** per group. (**b**) PERG latency was significantly higher in 4-week Roxa mice compared to control and 2-week Roxa mice (F(2,9) = 28.94, p = 0.0001). Tukey's multiple comparisons test showed PERG latency increased in 4-week Roxa compared to control (p = 0.0002) and 2-week Roxa (p = 0.0004) retinas. There were no significant differences in PERG latency between control vs. 2-week retinas (p = 0.7960). Values shown are mean ± SEM of **n = 4** per group. (**c**) Representative sample PERG traces from the Control, Roxa 2-week, and Roxa 4-week groups. (**d**)TUNEL-positive cell quantification in the GCL and INL of control and Roxa 4-week retinal cross-sections. There was no difference between control and 2-week Roxa GCL and INL for TUNEL + cell number, while 4-week Roxa treatment resulted in significant increases in GCL and INL when compared to control retina (p < 0.0001) and 2-week Roxa (p < 0.0001); two-way ANOVA with multiple comparisons, n = 6 retinas per group, with two fields per retina. (**e**) TUNEL staining (green) of retinal cross-sections from control and 2 and 4-week Roxa-treated mice. Nuclei stained with DAPI. (**f**) Quantification of RGCs in central and peripheral regions of control and 4-week Roxa-treated mouse retinas; n = 6 retinas per group, with two fields per retina. Control retina had significantly greater RGC number than central (p < 0.003) and peripheral (p < 0.0001) 4-week Roxa-treated central and peripheral retina, respectively; unpaired t-test. GCL = ganglion cell layer; INL = inner nuclear layer; ONL = outer nuclear layer. Scale bar: 20 μm. Data are shown as mean ± SEM.

Since we observed a significant change in the levels and activities of essential glycolytic and mitochondrial proteins, we evaluated the impact of chronic HIF-1α stabilization on RGC function and survival. For RGC function, we recorded pattern electroretinograms (PERG) in control and Roxa-treated mice. Average PERG amplitude was normal in 2-week Roxa mice but significantly reduced in 4-week Roxa mice compared to the control. PERG amplitude was significantly lower in 4-week Roxa compared to 2-week Roxa treatment mice (Fig. 8a). We observed a significant increase in PERG latency in 4-week Roxa mice compared to control and 2-week Roxa mice. However, there were no significant differences in PERG latency between control and 2-week Roxa mice (Fig. 8b). Representative traces from the PERG are shown in Fig. 8c. Degeneration of neurons is a common cause of visual impairment. To investigate whether prolonged stabilization of HIF-1α causes neuronal death, we performed terminal deoxynucleotidyl transferase (TdT) dUTP nick-end labeling (TUNEL) labeling on retinal cross-sections. Figure 8d shows quantification of the TUNEL + cells in retinal sections from the control, 2-week Roxa, and 4-week Roxa groups. By 4 weeks of Roxa treatment, there were significantly greater numbers of TUNEL + cells in the INL (p < 0.0001) and the GCL (p < 0.0001) of the retina when compared to control and 2-week Roxa (Fig. 8d). TUNEL + cells were prominent in the retina of 4-week Roxa mice, primarily located in the inner nuclear and ganglion cell layers (Fig. 8e). Quantification of RGCs showed a significant decline in RGC number for the 4-week Roxa treatment, in both central (p = 0.003) and peripheral (p < 0.0001) retinas compared to control (Fig. 8f).

## Discussion

Stabilization of HIF-1α protein is critical to maintaining energy generation in cells during hypoxia^12^. HIF-1α protein has been reported in the retina and ON from glaucoma patients and animal models^2,4,6,40–43^, indicating that increased IOP and potentially other mechanisms such as inflammation shift retinal metabolism towards glycolysis, including in the RGCs. However, the metabolic dysfunction in the glaucomatous retina suggests that the HIF-associated shift to glycolysis is ultimately inadequate to meet the metabolic needs of a functional retina. In addition, extensive HIF activation, including in the presence of oxygen, is detrimental to mitochondrial management because of the breakdown between nuclear and mitochondrial signaling in pseudohypoxia^14,17^. In the present study, we asked whether the repeated metabolic reprogramming that emerges from HIF activation in the absence of OHT mimics pseudohypoxia and can induce metabolic dysfunction and/or neurodegeneration.

We used a non-specific pharmacological inhibitor of oxygen-dependent PHDs to promote the stabilization of HIF-1α proteins in vivo under normoxia. Our results show that chronic HIF stabilization impaired the electron transport chain (ETC) as measured by significant reductions in succinate dehydrogenase A (SDHA), mitochondrial cytochrome c oxidase subunit 1 (MTCO1), and citrate synthase activity. Chronic HIF stabilization also increased pyruvate dehydrogenase kinase 1 (PDK1), BNIP3 dimer and BNIP3L/NIX, consistent with previous reports^44,45^. In contrast, we observed that prolonged HIF stabilization did not uniformly upregulate the expression of glycolysis pathway proteins. HIF stabilization increased neuron-specific glucose transporter (GLUT3) but significantly downregulated GLUT1, LDHA, and MCT4 protein levels. We also did not find significant changes in hexokinase activity after prolonged HIF-1α stabilization. Chronic HIF-1α stabilization led to AMPK activation, reactive gliosis, impaired visual function, and neurodegeneration. Our observations suggest a disconnect between chronic HIF-1α protein stabilization and expression of some HIF target genes in the retina similar to pseudohypoxia^17^. It also demonstrates that chronic HIF-1α stabilization can induce metabolic stress and neurodegeneration in the retina.

In general, HIF-1α stabilization upregulates the transcription of several glycolytic genes, notably glucose transporter 1 (GLUT1), glucose transporter 3 (GLUT3), hexokinase 2 (HK2), and lactate dehydrogenase A (LDHA)^12,46^. In Müller glia with acute Roxa treatment, we documented increases in glycolysis yet a decrease in overall glycolytic capacity. How Müller glia use glucose is up to debate, with indications that they do not possess the machinery to undergo glycolysis^47^, and that Müller glia use glucose primarily for serine production for photoreceptor support in the retina^48^ . However, Müller glia have been shown to engage in glycolysis^49,50^ , with stable isotope tracing showing ^13^C_6_-glucose resulting in ^13^C-pyruvate feeding into the TCA cycle in the human Müller MIO-M1 cell line^51^. In addition, scRNAseq data from mouse Müller glia show transcripts for glycolysis enzymes^52^. The various controversies regarding Müller glia metabolism notwithstanding, we were able to demonstrate that Roxa treatment increased glycolysis in mouse Müller glia in vitro*,* likely as a result of HIF-1α stabilization. In chronic glaucoma models, despite the evidence of increased HIF-1α in the retina, decreased GLUT1 and higher glucose levels have been reported^14,15^. Similarly, prolonged exposure to hypoxia did not alter LDHA expression in the brain of rats housed in a high-altitude environment^53^. It may be that the limited expression of HIF-1α targets in the retina during chronic hypoxia can be traced to the varied response of cells to PHD inhibition by Roxadustat. Additionally, our protein analysis used whole retinal lysate, thereby diluting any response variation across different cells.

Regulation independent of PHDs may explain the incomplete upregulation of glycolysis-associated HIF target genes. PHDs suppress HIF-1α at the protein level through oxygen-dependent prolyl hydroxylation^12^. However, the catalytic activity of PHDs is controlled by several cofactors, inhibitors and post-translational modifications (PTMs) that impede the association of HIF and its cofactors. One such PTM is the hydroxylation of asparagine 803 (Asp803) of HIF-1α by Factor inhibiting HIF-1 (FIH-1), an asparagine hydroxylase^54–56^. HIF-1α and HIF-2α have two transactivation domains, a C-terminal transactivation domain (C-TAD) and an N-terminal transactivation domain (N-TAD). Both N-TAD and C-TAD recruit coactivators for transcription induction, albeit only direct connections between the C-TAD and Creb-binding or p300 protein (CBP/p300) have been shown. Hydroxylation of Asp803 inhibits transcription by preventing the interaction of the C-terminal trans-activation domain (C-TAD) of HIF-1α with CBP/p300^54^. Most HIF target genes, but not all, are partially regulated by C-TAD. However, specific HIF target genes are solely N-TAD-dependent and unaffected by changes in C-TAD activity^57^; this indicates that the two HIF transactivation domains have overlapping but distinct functions. Since Roxadustat inhibits only PHDs, it follows that FIH-1 was still active and thus repressed C-TAD-mediated transactivation of HIF target genes. Interestingly, several glycolysis pathway proteins, including GLUT1, hexokinase 2 (HK2), and LDHA, that are associated with HIF-1α stabilization are inhibited by FIH-1 overexpression or induced exclusively by HIF-1α^58^. The downregulation of MCT4 we observed likely resulted from decreased glucose metabolism or a mechanism independent of glucose metabolism but mediated by chronic HIF stabilization. However, the expression of BNIP3, another C-TAD-regulated gene product, was upregulated. BNIP3 is a transcriptional target of HIF-1 that promotes adaptation to hypoxia by targeting mitochondria for degradation via autophagy (mitophagy). Consistent with HIF-1α stabilization and BNIP3 upregulation, we observed significant increases in mitophagy in both RGCs and Müller glia. BNIP3 regulates mitophagy via induction of mitochondrial fragmentation and direct interaction with the microtubule-associated protein 1A/1B-light chain 3 (LC3)^36,59^. Gene-specific variations in C-TAD-mediated HRE transactivation may have accounted for this observation. Thus, the extent to which C-TAD transactivation influences HIF target gene expression requires further study.

Although HIF-1α and HIF-2α protein stabilization often upregulates the transcription of HIF-targets, increasingly, acute versus chronic hypoxia exposure has been shown to result in different biological outcomes. For instance, compared to chronically hypoxic tumor cells, tumor cells exposed to acute hypoxia were more sensitive to radiotherapy^60,61^. This has largely been attributed to the temporal expression patterns of the HIF-α isoforms, HIF-1α and HIF-2α, with HIF-2α being the predominant isoform during chronic hypoxia^62^. Known regulators of differential expression HIF-α isoforms under prolonged hypoxia include the hypoxia-associated factor (HAF), which induces HIF-1α degradation but promotes HIF-2α transactivation, and the heat-shock protein 70/ carboxyl terminus of Hsp70-interacting protein (Hsp70/ CHIP) complex, which degrades HIF-1α but not HIF-2α^63–65^. Both HIF-1α and HIF-2α are deacetylated by Sirtuin 1(SIRT1). Sirtuins are NAD + -dependent protein deacetylases and ADP-ribosyl transferases that have been shown to regulate metabolism and aging^66,67^. Previous studies have shown that deacetylation by SIRT1 results in a decrease in HIF-1α transcriptional activity^68^ and an increase in HIF-2α in transcriptional activity^69^. In turn, it was discovered that both HIF-1α and HIF-2α under hypoxia increased SIRT1, which raises the possibility that SIRT1 may be involved in the negative- and positive-feedback loops that control HIF activity^70^. Additionally, HIF-2α can be stabilized and activated at higher oxygen concentrations than HIF-1α because it is hydroxylated by the PHDs and FIH-1 less efficiently^71^.

Although both HIF isoforms bind to HRE consensus sequences and control the expression of overlapping HIF-target genes, each isoform also has distinct target genes. In general, HIF-1α regulates the expression of glycolysis pathway genes such as HK1 (hexokinase 1), HK2 (hexokinase 2), and LDHA (lactate dehydrogenase A), whereas HIF-2α regulates genes involved in angiogenesis, including EPO (erythropoietin) and ANGPT2 (angiopoietin 2)^57,72–74^. Interestingly, Roxadustat is used clinically to combat anemia because of its ability to upregulate erythropoietin^75^. HIF-1α promotion of erythropoietin requires hypoxia independently of HIF-1α expression, suggesting that erythropoietin was not induced in the Roxa retinas^76^. Since erythropoietin has been shown to be neuroprotective for RGCs in the context of glaucoma^77^, our results showing significant apoptotic cells after 4 weeks of Roxa treatment also argue against erythropoietin induction. Furthermore, the Repressor Element 1-Silencing Transcription factor (REST), a transcription factor that binds to the HIF-1α promoter, was shown to inhibit HIF-1-dependent transcription, particularly of genes involved in glycolysis, during prolonged hypoxia in vitro^78^. Recent studies from our lab demonstrated a possible correlation between mitochondrial dysfunction, transcription failure, and pseudohypoxia glaucoma in DBA/2J mouse retina^14^. In another study where we used an acute mouse model of OHT, we showed that increased expression of HIF-2α mRNA did not alter GLUT1 levels^4^. However, whether FIH-1 and HIF-2α activities contribute to the metabolic decline in the glaucomatous retina is yet to be determined.

During hypoxia, activation of HIF can lead to mitochondrial dysfunction by altering the expression of mitochondrial proteins involved in the TCA cycle and the ETC^7,12^ while in pseudohypoxia, a breakdown of nuclear-mitochondrial signaling impairs the expression of nuclear-DNA encoded OXPHOS genes^79^. We show significant declines in ETC proteins SDHA and MTCO1 and citrate synthase activity following chronic HIF-1α stabilization. While the decrease in citrate synthase activity could have resulted from the accumulation of HIF target PDK1, the declines in SDHA and MTCO1 may have resulted from the increased mitophagy that we documented in RGCs and Müller glia. Our observation that BNIP3, a hypoxia-inducible protein that targets mitochondria for autophagy (mitophagy), was elevated supports this conclusion^38,80^. BNIP3 triggers mitophagy by causing the Bcl-2-Beclin-1 complex to disassemble, recruiting Parkin, an E3 ubiquitin ligase, to the mitochondria or directly binding to LC3 on the autophagosome^36,59,81^. Though BNIP3-mediated mitophagy during hypoxia is predicted to promote cell survival by preventing ROS accumulation from dysfunctional mitochondria, it has also been implicated in hypoxia-induced apoptosis^37^. PDK1, a mitochondrial protein, remained elevated; in contrast, other mitochondrial proteins decreased. We speculate that PDK1 may have accumulated outside the mitochondria as differential subcellular localization has previously been reported for various PDK isoforms^82^.

AMP-activated protein kinase (AMPK) is critical for regulating cellular energy homeostasis^83^. We found that AMPK was activated (phosphorylated) in response to chronic HIF-1α stabilization, consistent with the differential expression of glycolytic proteins and downregulation of ETC proteins. Thus, AMPK activation indicates that chronic HIF-1α stabilization impairs ATP generation in the retina. Upregulation of GFAP (reactive gliosis) occurs in glaucoma^84^ and multiple retinal diseases^85–87^; it is often used as a marker for retinal injury. Therefore, another sign of chronic HIF-1α induced stress was the enhanced expression of GFAP in 4-week Roxa-treated retinas. Short-term reactive gliosis is reported to be neuroprotective, whereas advanced gliosis has been linked to cell death^88^.

It is well established that RGC loss in glaucoma leads to reduced PERG amplitudes and increased latency periods^89,90^. However, there is no direct evidence of neurodegeneration resulting from hypoxia and HIF activation in the retina. In this study we showed a significant increase in apoptotic RGCs and inner retinal neurons following chronic stabilization of HIF by pharmacological inhibition of PHDs. Furthermore, we demonstrated chronic stabilization of HIF led to downregulation of glutamine synthetase (GS), which is a glial-specific protein that inhibits neurotoxicity by removing and recycling glutamate. RGC death may have resulted from chronic HIF activation-induced low energy status and a reduction in the neuroprotective function of GS.

In summary, these data provide evidence that chronic HIF-1α and HIF-2α activation, a feature of glaucoma and several retinal diseases, can induce metabolic dysfunction and neurodegeneration. Hence, our findings suggest that vision loss could be a potential adverse effect of the prolonged systemic use of PHD inhibitors such as Roxadustat in treating chronic kidney disease.

## Methods and materials

### Animals

All experimental procedures were approved by the University of North Texas Health Science Center Institutional Animal Care and Use Committee (protocols 2019–0020 and 2022–0026) and conformed to the Association for Research in Vision and Ophthalmology (ARVO) statement for use of animals in ophthalmic and vision research. The study is reported in accordance with ARRIVE guidelines. Both sexes of 2-month-old C57BL/6, CAG-creERT2-ODD, and B6.Cg-Gt(ROSA)26Sor^tm9(CAG-TdTomato)Hze^/J mice (Jackson Laboratory Stock #: 000664, MGI ID:5749962, and Jackson Laboratory Stock # 007909, respectively) were used in these experiments. The CAG-creERT2-ODD mice were crossed with the B6.Cg-Gt(ROSA)26Sor^tm9(CAG-TdTomato)Hze^/J to generate mice whose cells with stabilized HIF-1α are labelled with the tdTomato fluorescent protein following tamoxifen injection (Sigma-Aldrich T5648, 10 mg/kg in sunflower seed oil, IP once per day for 5 consecutive days). Both sexes of B6.Cg-Gt(ROSA)26Sor^tm9(CAG-TdTomato)Hze^/J, “MitoQC” mice (MRC Harwell Institute in Oxfordshire, UK) were used to assess mitophagy in this experiment. The MitoQC mice have an mCherry-GFP tag fused to the outer mitochondrial membrane, which allows the mitochondrial to fluoresce green and red under steady-state conditions^91^. These transgenic mice allow us to quantify mitophagy because the acidic pH within the lysosome quenches the green GFP fluorescence, leaving behind only the red mCherry fluorescence which is then counted as red puncta within the cytoplasm (see protocol below). Mice were fed ad libitum and kept on a 12:12 light/dark cycle.

### Intraocular pressure measurement

Mice were lightly anesthetized using isoflurane (~ 2%) and placed on an elevated platform from which a TonoLab rebound tonometer (iCare, Finland) was used to measure intraocular pressure 10 times from each eye. Eye sequence was varied to limit bias across left and right eyes. Values were averaged and presented as mean ± SEM mmHg; see Supplementary Fig. 1 online.

### Cell culture

Müller glia were isolated from 1-month-old C57Bl/6 mice. Dissected retinas were digested in papain with 200 U/mL of DNase (Worthington Biochemical, Lakewood, NJ) and incubated at 37 °C for 10 min. Next, an equal volume of ovomucoid (Worthington Biochemical) was added and retinas were gently triturated to obtain small pieces (~ 1 mm x 1 mm). After centrifugation at 300 × g for 10 min, tissue pieces were cultured in DMEM supplemented with 10% FBS (Sigma Aldrich, St. Louis, Missouri, United States) and 1% penicillin–streptomycin (Invitrogen, Carlsbad, California, United States). The media was changed every 3 days. Cells were maintained in culture for 8 weeks before analysis.

### Seahorse bioanalysis

Müller glia were plated in 24-well Seahorse plates, grown to ~ 80 percent confluency, and treated for 48 h with Roxadustat (10 µg/mL). The glycolytic stress test was undertaken on three separate Müller glia isolations using the Seahorse XFe24 Bioanalyzer (Agilent, Santa Clara, CA). After two washes of the cells in DMEM assay media, cells were incubated at 37 °C with room air. For the glycolytic stress test, a baseline oxygen consumption rate (OCR) and extracellular acidification rate (ECAR) were measured 3x, then the cells were exposed sequentially to glucose (10 mM), the ATP-synthase inhibitor oligomycin-A (1 µM), then the non-hydrolyzable 2-deoxyglucose (50 mM). After each new exposure, the OCR and ECAR were measured. For the fuel flexibility test, we explored the dependence and flexibility of Müller glia to use pyruvate first by inhibiting the mitochondrial pyruvate carrier with UK5099 and measuring OCR. After six OCR measures, the cells were exposed to inhibitors of both glutaminase (BPTES) and carnitine palmitoyl-transferase 1A (Etomoxir), with continued OCR measurement. Dependency of Müller glia on pyruvate was then calculated as the baseline OCR after subtracting the OCR from UK5099 exposure, divided by the baseline OCR after subtracting the OCR from BPTES and Etomoxir inhibition; the value is then multiplied by 100 to generate a dependency percentage. Capacity to use pyruvate by Müller glia was calculated by subjecting cells to Etomoxir and BPTES, followed by UK5099. The OCR of the BPTES + Etomoxir was subtracted from the baseline OCR, then divided by the OCR from the UK5099 addition subtracted from baseline OCR. This value, when subtracted from 1 and then multiplied by 100 is the capacity percentage. Flexibility is the dependency percentage subtracted from the capacity percentage. The fuel flexibility was run with 40 wells across two biological replicates.

### HIF stabilization

Mice were randomized into three groups and given the prolyl hydroxylase inhibitor Roxadustat (Cayman Chemicals, FG-4592); 1 mg/ml in PBS was administered to 2-month-old C57BL/6 mice by intraperitoneal injection at 10 mg/kg every other day for 2- or 4 weeks. Dose was determined by consensus from the literature for the IP route^92^. MitoQC mice were injected as described above every other day for 4 weeks. CAG-creERT2-ODD mice crossed with the B6.Cg-Gt(ROSA)26Sor^tm9(CAG-TdTomato)Hze^/J mice were dosed once with Roxadustat, 3 days prior to sacrifice. The European Medicines Agency risk management plan for the patient formulation of Roxadustat (EVRENZO), prescribed for the treatment of chronic kidney failure, includes thrombotic vascular events as important identified risks. Control mice (n = 2) and those treated with Roxadustat for 2 weeks (n = 2) and 4 weeks (n = 2) underwent fluorescein angiography to assess potential thrombosis in the retina. No indication of thrombosis was observed (Supplementary Fig. 2 online).

### Pattern electroretinography (PERG)

RGC function was analyzed using the binocular snout pattern ERG (PERG animal research system, Jörvec Corp., Miami, FL, USA). Mice were anesthetized (IP) with Ketamine (100 mg/kg)- Xylazine (10 mg/kg) and were kept on a heated stage during the procedure. Subcutaneous electrodes were placed in the mouse's snout (active), back of the head (reference), and tail (ground). PERG was concurrently obtained from each eye in response to the contrast reversal of gratings produced by two LED screens run at different frequencies. PERG data were evaluated by measuring amplitudes (N35-P50 and P50-N95) and corresponding implicit times (latencies). The average of two successive runs was used to plot the amplitudes and latencies.

### Perfusion and cryoprotection

Mice were euthanized at the end of 2 or 4 weeks with an overdose of sodium pentobarbital (300 mg/kg, Beuthanasia-D) then perfused transcardially with 0.1 M PBS, then with 4% PFA. Fixed eyes were cryoprotected in 0.1 M PBS containing 30% sucrose and 0.02% sodium azide and embedded in optimal cutting temperature (OCT) medium for sagittal sectioning at 8–10 mm using a Leica cryostat.

### Immunofluorescence (IF)

Sagittal sections of the retina were blocked in 5% donkey serum and 0.4% TritonX-100 in PBS for 1 h, then incubated with primary antibodies (listed in Table 1) overnight at 4 °C. After three washes (10 min each) in 0.1 M phosphate-buffered saline and a further 30 min block, secondary antibodies (1:250; Alexa-Fluor 488, 594, and 647; Jackson ImmunoResearch, West Grove, PA, USA) prepared in blocking medium were added, and incubated for 2 h at room temperature. Sections were rewashed in PBS, then cover-slipped using DAPI-Fluoromount-G (SouthernBiotech, Birmingham, AL, USA). TUNEL (Promega, Cat. #: 7130) assay was performed according to the manufacturer's instructions. We confirmed antibody specificity by incubating sections with secondary antibodies but without primary antibodies (Supplementary data, Fig. S3 online). Immunolabled tissue was imaged using a Zeiss LSM 880 AiryScan confocal microscope and resulting czi files were annotated using Adobe Photoshop.

**Table 1:** Antibodies used in immunofluorescence and capillary electrophoresis.

Antigen	Species	Manufacturer	Catalog number	Dilution
HIF-1α	Rabbit	Novus Biologicals	NB 100–134	1:50
GLUT1	Rabbit	Novus Biologicals	NB110-39,113	1:250 IF; 1:50 CE
GLUT3	Mouse	R&D Systems	MAB1415	1:25
LDH-A	Rabbit	Novus Biologicals	NBP1-48,336	1:50
MCT4	Mouse	Santa Cruz	SC-376465	1:50
MTCO1	Mouse	Invitrogen	459,600	1:100
SDHA	Mouse	Abcam	ab-14715	1:100
CRALBP	Mouse	Novus Biologicals	NB 100–74,392	1:200; 1:50 CE
GFAP	Goat	Abcam	ab53554	1:500
GLAST	Rabbit	Abcam	ab416	1:200 IF; 1:50 CE
Glutamine Synthetase	Mouse	Santa Cruz	SC-74430	1:150
Vimentin	Chicken	Novus Biologicals	NB300-233	1:250 IF
BNIP3	Rabbit	Cell Signaling	3769S	1:50
PDK1	Rabbit	Cell Signaling	C47H1	1:50
pAMPK (Thr 172)	Rabbit	Novus Biologicals	NBP1-7450	1:50
AMPK	Rabbit	Novus Biologicals	NBP2-22,127	1:50
RBPMS	Rabbit	GeneTex	GTX118619	1:250 IF

IF = immunofluorescence; CE = capillary electrophoresis.

### Protein analysis by capillary-based electrophoresis

Retinal proteins were extracted with T-PER buffer containing 1% HALT protease and phosphatase inhibitors (Thermo Fisher Scientific). Total protein concentration was estimated using the Bicinchoninic Acid Assay Kit (Pierce) and a Cytation 5 (Biotek) plate reader and analyzed by capillary tube-based electrophoresis immunoassay using the ProteinSimple Jess instrument. Each protein was normalized to the total protein in the sample capillary. Antibodies used for protein analysis are listed in Table 1.

### Biochemical assays

Retinas were isolated, flash-frozen in liquid nitrogen, then homogenized in assay buffer. Tissue homogenates were centrifuged at 10,000 g for 10 min at 4 °C, and then the supernatant was collected and kept at − 80 °C until ready to use. All the biochemical assays were performed according to the manufacturer's instructions (see Table 2). Biochemical data were normalized by the total protein level of each retina sample. Retina total protein was quantified using the Pierce™ BCA protein assay kit.

**Table 2:** Biochemical assay kits.

Parameter	Manufacturer	Catalog number
Hexokinase	Millipore-Sigma	MAK091-1KT
Citrate Synthase	Millipore-Sigma	CS0720-1KT

### Mitolysosome quantification

Slides were stained as described in the Immunofluorescence section with either RBPMS for RGCs or Vimentin for Müller glia to assess mitophagy in both cell types. Images for analysis were generated by capturing 2 images/retinal section for 6 retinal sections for each eye per mouse on the Zeiss LSM 880 with Airyscan Confocal microscope. Z-stack images were taken at 63X magnification. A FIJI-Image J macro called “MitoQC Counter” was used with guidance from Lambert, et al.^93^ for image preprocessing prior to quantification using mQC_counter. In brief, within ImageJ, image color channels were split, selecting the channel representing either RBPMS or Vimentin for adjustment of brightness/contrast. Under the Process tab, there is an option for filters; median was selected, with the radius set to 5.0 pixels. After thresholding (also under the Adjust option) to ensure the threshold covers the stained cells, Binary option and Watershed (Process tab) were selected to separate joined cells when necessary. Next, Analyze particles was selected and set size to 0.01—infinity. Channels were merged under the Color option, specifying C1 as the red channel image, C2 as the green channel image and C3 as the blue channel image. In the ROI window, Show All was specified. The mQC_counter option (Plugins) was selected, setting green channel as 2 and red channel as 1 and ratio threshold as 0.5. The output from the mQC_counter plugin was then normalized by cell size and presented as mitolysosomes per cell area in µm^2^.

Images in Fig. 6 of mitochondria and mitolysosomes within RGCs and Müller glia were individual confocal images taken with a Zeiss LSM 880 then subjected to deconvolution using Huygens software from Scientific Volume Imaging.

### Statistics

Data were analyzed using GraphPad Prism v.9 (La Jolla, CA, USA).

Statistical analyses were performed after evaluating normality. When distributions were Gaussian, One-way ANOVA and Tukey's multiple comparison post hoc test were used to compare differences across groups within an outcome measure; p < 0.05 was considered statistically significant, and data are reported as mean ± SEM. Two-way ANOVA with multiple comparisons was used to compare retinal layer by experimental group for the TUNEL + cell quantification.

### Supplementary Information

Supplementary Information.

## Data Availability

All data generated or analyzed for this investigation are included in this publication.
