# Supplementary material for: Long-term HIF-1α stabilization reduces respiration, promotes mitophagy, and results in retinal cell death
Source: Sci Rep. 2023 Nov 23;13:20541. doi: 10.1038/s41598-023-47942-8 (PMC10667534; doi:10.1038/s41598-023-47942-8)

Supplementary Data

**Long-term HIF-1α Stabilization Reduces Respiration, Promotes Mitophagy, and Results in Retinal Cell Death**

Nana Yaa Nsiah, Autumn B. Morgan, Nina Donkor, Denise M. Inman

Supplementary Figure 1.


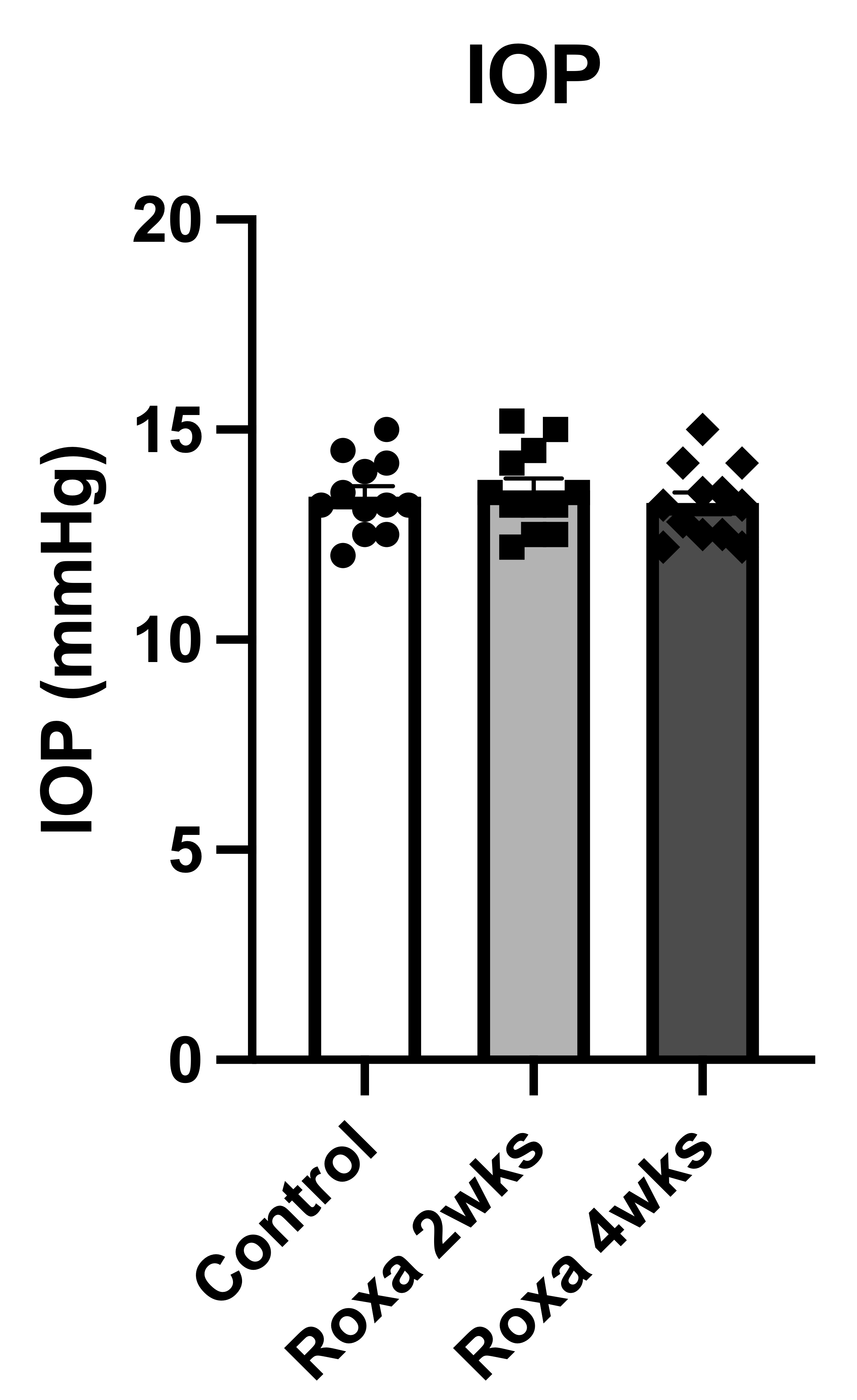


Intraocular pressure measurements showed no statistical difference among Control mice and those dosed with Roxadustat for 2 or 4 weeks (p=0.714 by one-way ANOVA).

| **Group** | **IOP ± SEM (mmHg)** |
| --- | --- |
| Control | 13.41 ± 0.255 |
| Roxa 2 Weeks | 13.56 ± 0.283 |
| Roxa 4 Weeks | 13.25 ± 0.254 |

Supplementary Figure 2.


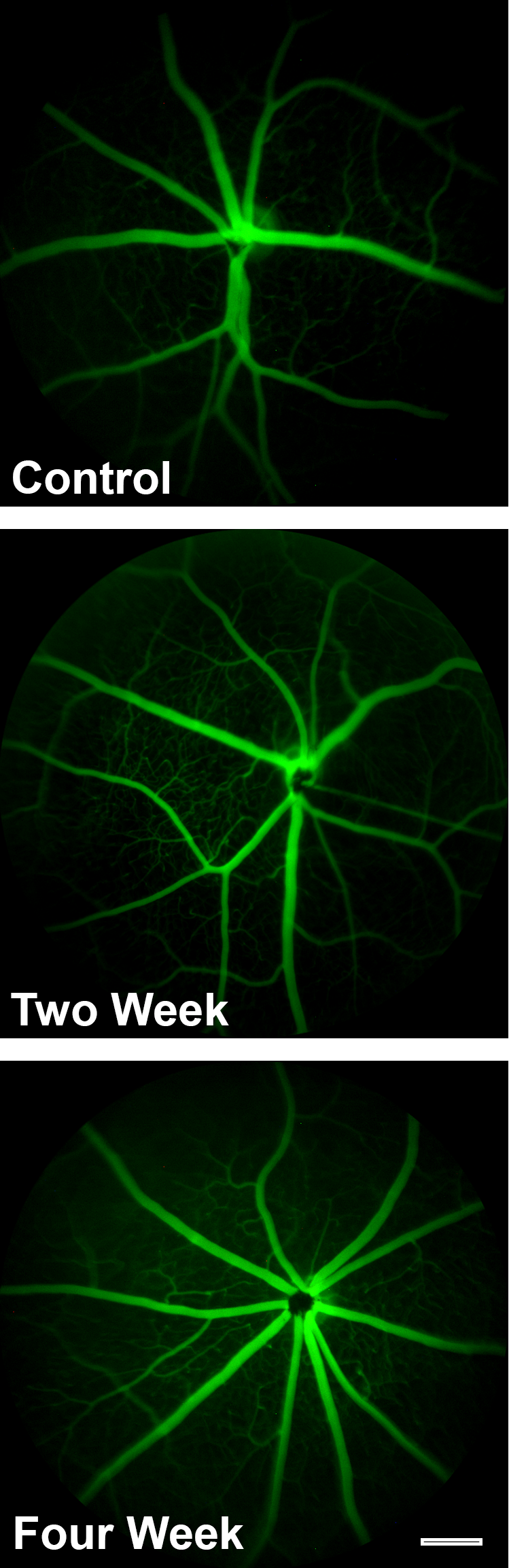


Two mice per group—Control, 2-week Roxadustat treatment, and 4-week Roxadustat treatment—were anesthetized with 2.5% isoflurane, had their pupils dilated with topical 0.5% Tropicamide, and then received an IP injection of 1% fluorescein in 0.1M PBS. Eyes were then covered in GenTeal Tears lubricant while the mouse was positioned for imaging. Within 3-5min, fluorescein was detected in the right eye, and an image was taken using the Micron IV (Phoenix) imaging system. An image was also taken of the left eye for each mouse. The photomicrograph shows an example image from one eye for each of the groups. Scale bar=500μm.

Supplementary Figure 3.

No primary antibody controls.

**Anti-Rabbit AlexaFluor 488 [Figures 1C, 3D, 4D, 8E]**


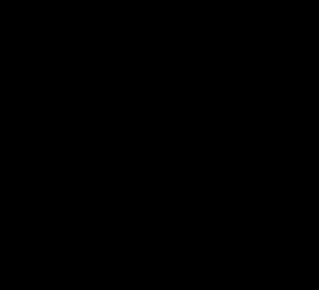
AF488 and DAPI AF488 alone


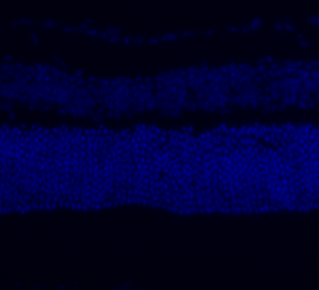


**Anti-Rabbit AlexaFluor 647 [Figures 1C, 5B, 6A]**

AF647 and DAPI AF647 alone


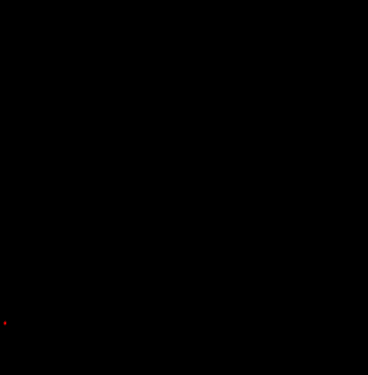

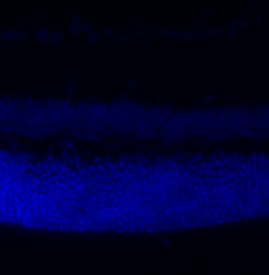


**Anti-Mouse AlexaFluor 647 [Figure 3, 4D, 7B]**

AF647 and DAPI AF647 alone

**
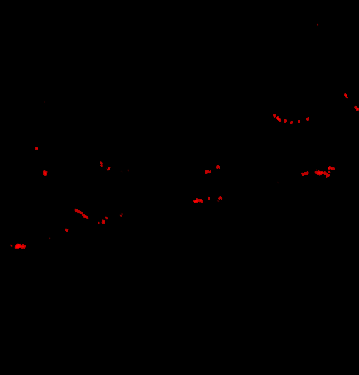

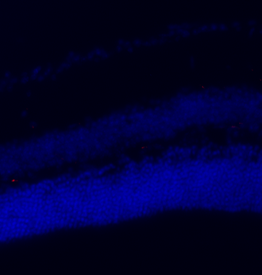
**

**Anti-Mouse AlexaFluor 488 [Figure 5B]**

AF488 and DAPI AF488 alone

**
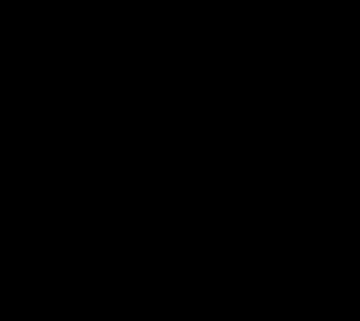

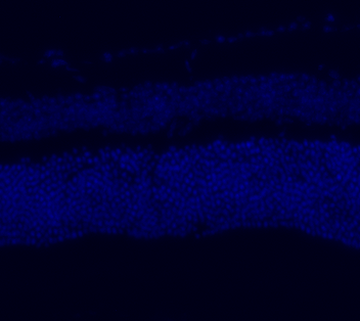
**

**Anti-Goat AlexaFluor 488 [Figure 7D]**

AF488 and DAPI AF 488 Alone

**
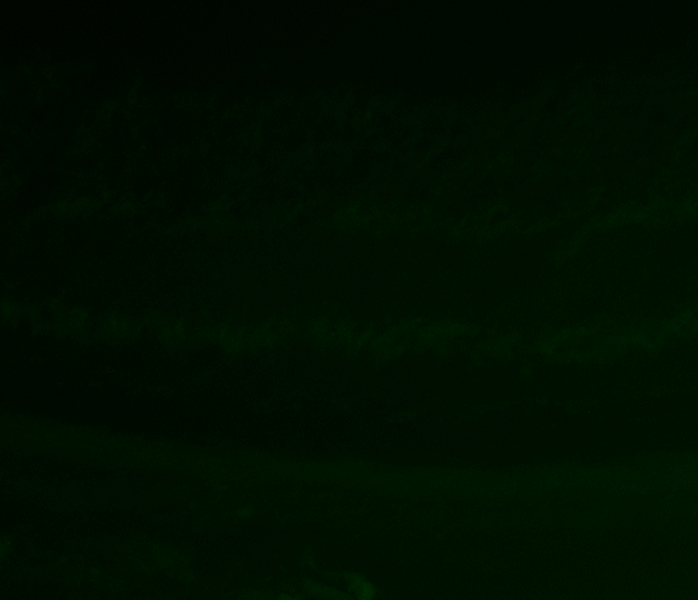

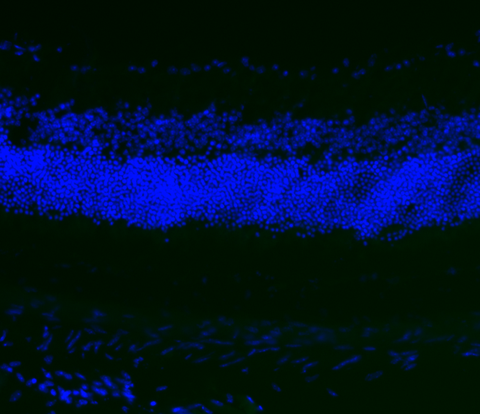
**

**Anti-Chicken AlexaFluor 594 [Figure 7D]**

AF594 and DAPI AF594 Alone


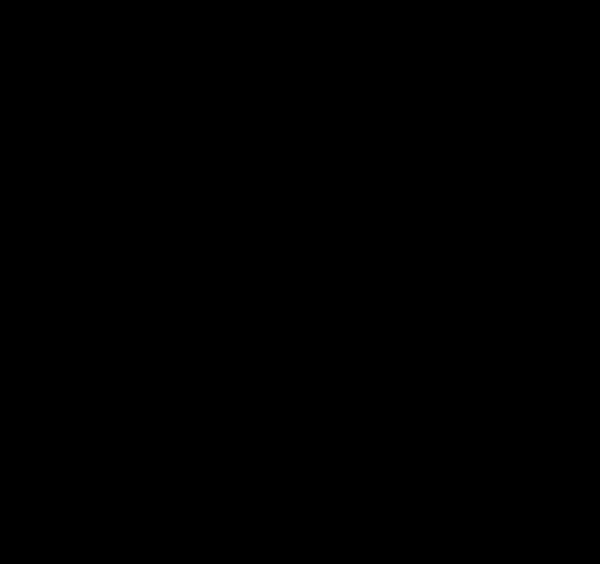

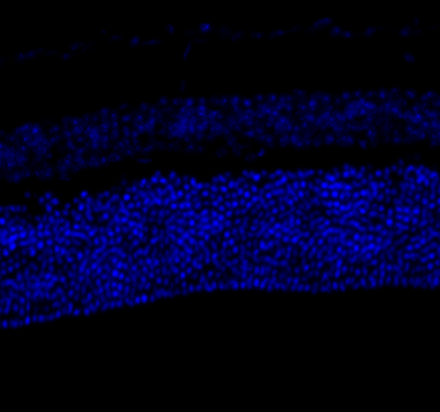

Supplement: Supplementary file 1 — Supplementary Information. [file 41598_2023_47942_MOESM1_ESM.docx]
